# Does feeling respected influence return to work? Cross-sectional study on sick-listed patients’ experiences of encounters with social insurance office staff

**DOI:** 10.1186/1471-2458-13-268

**Published:** 2013-03-23

**Authors:** Niels Lynöe, Maja Wessel, Daniel Olsson, Kristina Alexanderson, Gert Helgesson

**Affiliations:** 1 Stockholm Centre for Healthcare Ethics (CHE), Department of Learning, Informatics, Management and Ethics, Karolinska Institutet, Stockholm SE-171 77, Sweden; 2 Department of Environmental Medicine (IMM), Unit of Biostatistics, Division of Epidemiology, Karolinska Institutet, Stockholm, Sweden; 3 Department of Clinical Neuroscience (CNS), Division of Insurance Medicine, Karolinska Institutet, Stockholm, Sweden

**Keywords:** Encounters, Ethics, Long-term sickness absentees, Return to work, Social insurance office staff, Sweden

## Abstract

**Background:**

Previous research shows that how patients perceive encounters with healthcare staff may affect their health and self-estimated ability to return to work. The aim of the present study was to explore long-term sick-listed patients’ encounters with social insurance office staff and the impact of these encounters on self-estimated ability to return to work.

**Methods:**

A random sample of long-term sick-listed patients (n = 10,042) received a questionnaire containing questions about their experiences of positive and negative encounters and item lists specifying such experiences. Respondents were also asked whether the encounters made them feel respected or wronged and how they estimated the effect of these encounters on their ability to return to work. Statistical analysis was conducted using 95% confidence intervals (CI) for proportions, and attributable risk (AR) with 95% CI.

**Results:**

The response rate was 58%. Encounter items strongly associated with feeling respected were, among others: listened to me, believed me, and answered my questions. Encounter items strongly associated with feeling wronged were, among others: did not believe me, doubted my condition, and questioned my motivation to work. Positive encounters facilitated patients’ self-estimated ability to return to work [26.9% (CI: 22.1-31.7)]. This effect was significantly increased if the patients also felt respected [49.3% (CI: 47.5-51.1)]. Negative encounters impeded self-estimated ability to return to work [29.1% (CI: 24.6-33.6)]; when also feeling wronged return to work was significantly further impeded [51.3% (CI: 47.1-55.5)].

**Conclusions:**

Long-term sick-listed patients find that their self-reported ability to return to work is affected by positive and negative encounters with social insurance office staff. This effect is further enhanced by feeling respected or wronged, respectively.

## Background

Ways of promoting return to work among sickness absentees is an ongoing clinical as well as political theme in many western countries, facilitating return to work among long-term sickness absentees being a special topic of concern. Several different interventions and programs have been introduced to this end, at different structural levels and among different stakeholders, e.g. worksites, healthcare, and insurance offices [1-12].

The long-term sick-listed constitute a vulnerable group in different ways: apart from their morbidity/health condition, they often have a lower educational level compared to others, are more often immigrants, and have a lower disposable income compared to their previous work income [13]. Moreover, to a large degree they are in the hands of both healthcare staff and social insurance office staff, whose judgments and decisions have a major impact on their access to rehabilitation measures, work adjustments, and their economic situation. Long-term sickness absentees have themselves stated that their treatment by professionals from healthcare and social insurance was as important as the different rehabilitation measures [14,15]. For instance, respectful meetings have been reported to be of relevance for returning to work [9].

In previously conducted studies regarding long-term sickness absentees’ encounters with healthcare staff, based on data from the same questionnaire as the present study uses, we found that both negative and positive encounters influence patients’ self-estimated ability to return to work [16]. We also found that when patients feel respected in addition to experiencing their encounters as positive, their self-estimated ability to return to work is significantly facilitated, whereas patients feeling wronged in addition to experiencing their encounters as negative, estimated that their ability to return to work was significantly impeded [17]. Even though self-estimated ability to return to work is not the same as actually returning to work, long-term sick-listed patients’ own beliefs on this matter is arguably an important predictor for return to work, in a similar vein as self-estimated health has been reported as the most valid predictor of a long life [9,18].

Since it is social insurance office staffs who decide whether patients fulfil the criteria for sickness benefits, it is also of interest to investigate patients’ perceptions of encounters with these actors. The aim of the present study was to explore long-term sick-listed patients’ encounters with social insurance office staff and the impact of these encounters on their self-estimated ability to return to work. To further illuminate these patients’ experiences of encounters with social insurance office staff, those experiences were compared with their experiences of encounters with healthcare staff.

## Methods

In this cross-sectional study we analysed data from answers to a population-based questionnaire sent out to a random selection of half of all people in Sweden who in March 2004 had an on-going sick-leave spell that had lasted for at least four and at the most eight months (n = 10,042). This duration of the sick-leave spell was chosen so that the absentees would have had a chance to have personal contact with the social insurance office but still have a good chance to return to work. A comprehensive questionnaire was developed, based on several qualitative and quantitative studies of how sickness absentees experience encounters with social insurance and health care staff [14,19-21].

We studied their experiences of positive and negative encounters with social insurance office staff. All participants received the same questionnaire. Those who had experienced positive encounters only were subsequently asked to specify the experience by choosing from a list of different positive encounters, such as ‘Listened to me’ and ‘Believed me’. They were also asked what kind of feeling the encounters had resulted in, including feeling respected. Those with experiences of negative encounters only or a mix of positive and negative encounters were similarly presented to a list of negative encounters, such as ‘Did not listen’ and ‘Was too impersonal’, and they were also asked what kind of emotions the encounters resulted in, including feeling wronged. Finally all participants were asked whether or not the encounters had influenced their ability to return to work, response alternatives being ‘facilitating’, ‘not influencing’, or ‘impeding’. The lists of encounter items were developed partly based on the outcome of focus-group interviews [19].

In addition, the respondents were asked if they were sick-listed due to (a) mental disorders, (b) musculoskeletal pain, or (c) other somatic diseases.

Focusing on the associations between feeling respected / wronged in encounters with social insurance office staff and self-estimated ability to return to work, we performed logistic regression analysis adjusting for different background variables such as sex, age, educational level, and diagnosis. Adjustments made no substantial difference to the results. Therefore, we present the results as proportions with a 95% confidence interval (CI) for those who estimated that return to work was facilitated when experiencing positive / respectful encounters compared to those who stated that it was not influenced or impeded. The same was done for the proportion of those whose self-estimated ability to return to work was impeded when exposed to negative/wrongful encounters compared to those who stated that it was not influenced or facilitated.

The associations between positive encounters and feeling respected, and negative encounters and feeling wronged, are presented as attributable risk (AR) with a 95% CI, using the R-package pARtial [22]. All ARs were adjusted for sex, age (20–29, 30–39, 40–49, 50–59, and 60–65 years), education (compulsory school, 2 years in high school, 3–4 years in high school, university credits, completed university degree), and reason for being sick-listed (Table 1). AR takes into account both frequency and strength of association in a certain population. AR for those who felt wronged in relation to specific encounter-items could be interpreted as: If social insurance staffs had, for example, listened to the patients, 39.8% would not have felt wronged, (Table 2). Results concerning return to work were presented as proportions with 95% CI. When comparing the results regarding social insurance office staff with results regarding healthcare staff, we included results that have been published elsewhere [17,23].

**Table 1:** Demographic presentation of the study population, responders, and of the sample population that had experienced negative encounters with social insurance staff

	**Total number of respondents n (%)**	**Respondents with experience of positive encounters n (%)**	**Respondents with experience of negative encounters n (%)**
**All**	5,802 (100)	4,365 (100)	1,206 (100)
*Gender*			
Female	3,698 (64)	2,811 (64)	790 (66)
Male	2,104 (36)	1,554 (36)	416 (34)
*Age categories*			
20-29	460 (8)	331 (8)	169 (14)
30-39	1,177 (20)	897 (20)	334 (28)
40-49	1,424 (25)	1,058 (24)	326 (27)
50-59	1,825 (31)	1,397 (32)	285 (24)
60-64	916 (16)	682 (16)	92 (7)
*Educational level*			
Compulsory school	1759 (30)	1324 (30)	287 (24)
2 years in high school	1251 (22)	950 (22)	272 (23)
3-4 years in high school	1171 (20)	883 (20)	299 (25)
University credits	490 (8)	386 (9)	105 (8)
Completed university degree	1074 (19)	786 (18)	233 (19)
Missing	56 (1)	36 (1)	10 (1)
*Sick-leave diagnosis*			
Mental	1,547 (27)	1161 (27)	335 (28)
Musculoskeletal	1,855 (32)	1422 (32)	386 (32)
Other somatic	1,322 (23)	964 (22)	218 (18)
Several diagnoses	1,069 (18)	256 (6)	133 (11)
(Missing)	9	562 (13)	134 (11)

**Table 2:** Positive encounters in terms of feeling respected among long-term sick-listed patients and their contact with social insurance office staff

**Type of positive encounter with: (n = social insurance office staff/healthcare staff)**	**Social insurance office staff**	**Healthcare office staff**
	**n = 4622**	**n = 5277**
	**AR (95% CI)**	**AR(95% CI)**
Treated me with respect (n = 2956/3223)	89.5% (87.6-91.4)*	80.2% (77.2-83.2)
Listened to me (n = 2950/3224)	89.0% (87.0-90.9)*	80.3% (76.7-83.9)
Nice/pleasant behaviour (n = 2942/3160)	87.9% (85.8-90.0)*	77.1% (73.4-80.6)
Believed me (n = 2913/3201)	85.5% (83.4-87.7)*	66.0% (62.2-69.7)
Answered my questions (n = 2820/2962)	78.0% (75.6-80.4)*	66.6% (62.8-70.3)
Was competent (n = 2677/2787)	64.5% (64.5-69.7)	61.8% (58.2-65.5)
Showed engagement (n = 2604/2754)	63.5% (60.9-66.1)*	55.5% (52.0-59.0)
Let me take responsibility (n = 2612/2806)	62.8% (60.2-65.4)*	38.6% (35.4-41.8)
Made reasonable demands (n = 2545/2599)	60.4% (57.9-62.9)*	46.9% (43.6-50.2)
Took time with me during our meetings (n = 2514/2492)	57.6% (55.1-60.1)	55.1% (51.4-58.8)
Believed in my ability to work (n = 2536/2751)	57.6% (54.9-60.2)*	27.2% (24.6-29.7)
Gave adequate information (n = 2371/2456)	51.8% (49.3-54.2)*	39.2% (36.0-42.4)
Defended me/was on my side (n = 2291/2293)	48.3% (45.9-50.6)*	38.3% (35.4-41.2)
Supported my suggestions for solutions (n = 2207/2254)	44.6% (42.3-46.9)*	33.4% (30.7-36.1)
Was easy to get an appointment with (n = 2134/2142)	41.5% (39.2-43.7)*	19.0% (17.1-21.0)
Was supportive and encouraging (n = 1951/1957)	36.0% (34.0-38.0)	33.4% (30.7-36.1)
Showed that he/she liked me (n = 1804/1744)	31.9% (30.0-33.7)*	25.9% (23.7-28.2)
Did something “extra” (n = 1451/1406)	22.5% (21.0-24.0)*	14.8% (13.4-16.2)
Talked about him-/herself (n = 697/669)	8.7% (7.9-9.5)*	5.6% (4.9-6.2 )

The results are compared to similar encounters with healthcare staff. Results are presented as population proportional attributable risk (AR) with 95% confidence intervals (CI). A * means that CIs were not overlapping.

The study was approved by the Regional Research Ethics Committee in Linköping, Dnr 03–261.

## Results

**Figure 1:**
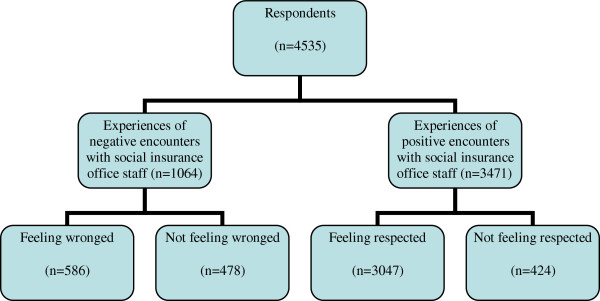
**Distribution of the long-term sickness absent population who had experienced negative or positive encounters with social insurance staff.** The respondents are divided into those who felt wronged and those who did not feel wronged, or felt respected or did not feel respected.

The response rate was 58% (n = 5,802) of the original sample (Table 1). When asked about experiences of negative and positive encounters, there was an internal drop-out of 231 ending up with 5,571 participants. Of these, 78.4% had experienced positive encounters and 21.6% had experienced negative encounters. When the respondents were asked about feeling respected or wronged, there was an additional internal drop-out, leaving 4,535 participants, of whom 76.5% had felt respected and 23.5% had felt wronged (Figure 1).

Of those participants who had experienced positive encounters, 87.8% (n = 3,047) reported that they had also felt respected. Of those who had experienced negative encounters, 55.1% (n = 586) reported that they had also felt wronged (Figure 1).

### Effects of positive encounters and feeling respected

We found a high attributable risk (AR) for having experienced positive encounters and the patient’s feeling respected. Being listened to and being believed were among the types of behaviour with the highest AR (Table 2).

Of those with experience of positive encounters, 26.9% (95% CI: 22.1-31.7) stated that it facilitated their self-estimated ability to return to work. This ability was significantly increased if they also felt respected [49.3% (47.5-51.1)]. In particular, patients with psychiatric disorders estimated that their ability to return to work was significantly improved when they felt respected (Table 3).

**Table 3:** Proportions of those who, following contact with social insurance office staff, reported that encounters facilitated return to work (with a 95 per cent confidence interval)

	**Experiences of encounters with social insurance office staff**	**Experiences of encounters with healthcare staff**
**(n = social insurance office staff/healthcare staff)**	**Return to work facilitated**	**Return to work facilitated**
*Mental diagnosis*		
Positive encounters (n = 580/1083)	28.1% (17.8-37.4)	37.5% (23.8-51.2)
Also felt respected (n = 501/924)	60% (56.7-63.3)*	76.3% (73.6-79)
*Chronic pain conditions*		
Positive encounters (n = 480/1269)	21.1% (13.6-28.6)	34.3% (23.4-45.2)
Also felt respected (n = 405/1036)	43.9% (40.7-47.1)*	52.7% (49.6-55.8)
*Somatic diagnosis*		
Positive encounters (n = 318/868)	22.2% (11.9-32.5)	27% (12.7-41.3)
Also felt respected (n = 254/667)	42.1% (38.2-46)*	54.0% (50.5-57.5)

A * means that CIs were not overlapping. The results are divided into the three main diagnoses: psychiatric disorders, musculoskeletal pain, and other somatic diseases. The results are compared to similar data from patient contacts with healthcare staff.

### Impact of negative encounters and feeling wronged

The risk of feeling wronged if exposed to negative encounters was found to be high. The highest AR was linked with being treated with nonchalance, being disbelieved, and having one’s condition doubted (Table 4).

**Table 4:** Negative encounters in terms of feeling wronged among long-term sick-listed patients and their contact with social insurance office staff

**Type of negative encounter with: (n = social insurance office staff/healthcare staff)**	**Social insurance office staff**	**Healthcare office staff**
	**n = 1206**	**n = 1628**
	**AR (95% CI)**	**AR (95% CI)**
Nonchalant behaviour (n = 508/1280)	61.1% (54.3-67.9)	71.1% (66.3-75.8)
Treated me with disrespect (n = 447/1041)	52.4% (46.3-58.0)	54.8% (49.8-59.8)
Did not believe me (n = 430/1042)	43.2% (37.2-49.1)	41.1% (36.1-46.1)
Doubted my condition (n = 421/1077)	42.2% (36.4-48.1)	36.8% (31.4-41.1)
Questioned my motivation to work (n = 423/913)	41.4% (35.2-47.6)*	23.9% (19.9-28.0)
Did not listen (n = 386/982)	39.8% (34.6-45.0)	34.6% (30.2-39.0)
Rejected my suggestions for solutions (n = 403/903)	39.8% (34.2-45.4)*	28.4% (24.3-32.4)
Was too impersonal (n = 406/916)	38.6% (33.1-39.7)	29.2% (24.9-33.4)
Treated me as stupid (n = 351/808)	34.9% (30.1-39.7)	32.5% (28.6-36.4)
Was irritated/ impatient (n = 380/914)	34.5% (29.2-39.8)	31.2% (26.9-35.4)
Angry/unpleasant behaviour (n = 315/706)	27.4% (22.9-31.8)	26.4% (23.1-29.8)
Interrupted me (n = 277/1022)	25.2% (21.2-29.2)	20.3% (17.1-23.4)
Made unreasonably high demands (n = 309/787)	24.7% (20.0-29.3)*	15.6% (12.0-19.2)
Was stressed/ did not make time for me (n = 348/1075)	23.9% (18.5-29.3)	24.9% (19.9-29.9)
Did not let me take responsibility for myself (n = 253/469)	21.2% (17.3-25.0)*	10.7% (8.4-13.0)
Blamed me for my condition (n = 186/451)	16.9% (13.9-19.9)	12.2% (10.0-14.4)
Did not keep our agreements (n = 221/418)	16.9% (13.2-20.4)*	6.5% (4.4-8.6)
Doubted my capacity to work (n = 315/693)	16.7% (11.5-21.9)	9.3% (6.2-12.4)
Talked in a way I could not understand (n = 188/397)	11.7% (8.2-15.2)*	4.5% (2.3-6.7)
Threatened me (n = 98/116)	7.7% (5.6-9.9)*	3.3% (2.4-4.2)
Did not make high enough demands (n = 52/117)	3.3% (1.6-5.0)	1.7% (1.2-2.6)
Harmed me physically (n = 31/103)	2.0% (0.8-3.3)	2.1% (1.2-3.0)
Sexually inappropriate behaviour (n = 17/30)	0.4% (−0.5-1.4)	0.7% (0.2-1.1)

The results are compared to similar encounters with healthcare staff. Results are presented as attributable risk (AR) with 95% confidence intervals. A * means that CIs were not overlapping.

Of those with experience of negative encounters, 29.1% (24.6-33.6) stated that these experiences impeded their ability to return to work. A significantly greater proportion of the respondents who in addition felt wronged described themselves as impeded from returning to work [51.3% (47.1-55.5)]. Compared to patients suffering from somatic disorders, more patients with mental disorders reported this effect when also feeling wronged (Table 5).

**Table 5:** Proportions of those who, following contact with staff of the social insurance office, reported that they were impeded from returning to work (with a 95% confidence interval)

	**Experiences of encounters with social insurance office staff**	**Experiences of encounters with healthcare staff**
**(n = social insurance office staff/healthcare staff)**	**Return to work impeded**	**Return to work impeded**
*Mental diagnosis*		
Negative encounters (n = 149/290)	36.1% (27.5-44.7)	38.5% (29.1-47.9)
Also feeling wronged (n = 93/268)	62.4% (54.6-70.2)	59.2% (53.8-64.6)
*Chronic pain conditions*		
Negative encounters (n = 114/332)	26.3% (18.2-34.4)	26.8% (19.5-34.1)
Also feeling wronged (n = 70/293)	39.1% (32–46.2)	43.7% (38.1-49.3)
*Somatic diagnosis*		
Negative encounters (n = 61/189)	23% (13.4-32.6)	27.9% (18.4-37.4)
Also feeing wronged (n = 40/176)	39.2% (29.7-48.7)	39.1% (31.6-46.6)

The results are divided into the three main diagnoses: psychiatric disorders, musculoskeletal disorders, and other somatic diseases. The results are compared to similar data from patient contacts with healthcare staff.

We found no significant differences based on sex or social status concerning the different aspects discussed above.

## Discussion

We found high ARs for positive encounters with social insurance office staff and feeling respected and for negative encounters and feeling wronged. We also found that feeling respected had a facilitating effect on a self-reported return-to-work ability. Similarly, we found that feeling wronged had an impeding effect on self-reportedly ability to return to work. The positive effect of feeling respected on self-estimated ability to return to work was particularly manifested among patients with mental disorders. Our results are in line with previous findings that long-term sick-listed patients are sensitive to whether their encounters are respectful or not [9].

Specific items of positive encounters particularly associated with feeling respected were being listened to, being believed in, and having one’s questions answered. Specific items of negative encounters associated with feeling wronged were not being believed, having one’s condition doubted, and having one’s motivation for work questioned. “Nonchalant behaviour” was the item of negative encounters with the highest AR, but it is highly unspecific and might cover several of the more specific items. The same goes for the item of positive encounters with the highest AR, “Treated me with respect”.

From other studies it has been reported that female sick-listed patients have special preferences when it comes to rehabilitation and return to work [9]. On this backdrop it is interesting that we did not find any gender differences. This difference might be due to different methods; it might be easier to recognize gender aspects in qualitative research compared to quantitative studies.

### Comparing encounters with social insurance office staff and healthcare staff

Our survey regarding encounters with social insurance office staff also collected data regarding patients’ encounters with healthcare staff, published elsewhere [17,23,24]. Regarding positive encounters and feeling respected, a majority of encounter items yielded significantly higher ARs among social insurance staff compared to healthcare staff. Also, negative encounters and feeling wronged displayed a tendency for social insurance staff to score higher ARs [17,23]. The few items which had significantly higher ARs are rather interesting. The long-term sick-listed seem to feel that it is worse if the staff at a social insurance office question their motivation to work, reject their suggested solutions, or threaten them. How can these differences be explained? Compared to healthcare staff, who are primarily concerned with patients’ health, social insurance office staff have other tasks associated with societal, economic, and regulatory interests. It is part of their job to assess their clients’ right to sickness benefit. Being questioned on this matter implies a threat to the income of the concerned individuals and can therefore become a very sensitive matter in that context. This might explain the attitude towards having one’s willingness to work questioned or one’s suggestions for handling the situation rejected. Perhaps the threats experienced concern financial actions that social insurance office staff might take if the client does not behave in accordance with requirements.

When comparing positive encounters associated with long-term sick-listed individuals’ feeling of being respected by social insurance office staff or healthcare staff, we identified several significant differences. The respondents stated that it is more important that social insurance office staff believe in them and in their ability to work, let them take responsibility, and make reasonable demands, compared to healthcare staff doing so. Again, these differences might be explained by the different roles of social insurance office staff and healthcare staff.

### Need to increase awareness of negative encounters

Patients might react differently to the same kinds of encounters, depending on their personal sensitivity and circumstances [12]. Sometimes healthcare staff and social insurance office staff might be provoked or intimidated by patients/clients and confrontations might occur [25]. Studies have indicated that it is unreasonable to assume that healthcare staff intentionally wrong patients [26]. Similar reasoning might be plausible when discussing social insurance office staff. Yet both professional groups need to become aware of what kinds of encounters are negatively experienced by patients and might cause them to feel wronged. Even if patients’ perceptions do not always correspond to objective negative or wrongful behaviour, these perceptions need to be taken seriously, because they seem to have consequences, for example effects on patients’ self-estimated ability to return to work. Apparently the quality of encounters is not solely a matter of etiquette.

### Different perceived effects on return to work

There were also differences in the encounters’ perceived effect on return to work. However, when comparing the two contexts, we found no significant difference regarding feeling wronged and self-estimated effect on return to work. Nevertheless, for positive encounters in which patients also felt respected, the behaviour of healthcare staff was more commonly perceived than that of social insurance office staff as facilitating return to work. Yet the impact in the latter case is not negligible, according to the respondents’ self-estimations.

Even without considering the consequences for return to work, it seems reasonable to promote encounters that facilitate individuals’ feeling of being respected and avoid encounters that make them feel wronged. When this aspect is also considered, the reasons become even stronger. From the perspective of positive encounters and feeling respected, social insurance staff ought particularly to avoid questioning patients’ work morality, rejecting their suggested solutions, making unreasonable demands, and threatening the patient.

### Limitations

The present study was a cross-sectional study, with its inherent limitations. Although the response rate was relatively high, there was also an internal drop-out that should be taken into consideration. The internal dropout rate increased in every combined step (Figure 1).

The focus was on long-term sick-listed patients’ encounters with social insurance office staff and the self-estimated effects of these encounters on return to work. Although self-estimated work-capacity might influence actual capacity, self-estimated effects and actual effects on return to work are distinct entities. The results should therefore be interpreted carefully about the effects of positive and negative encounters on actual return to work. Furthermore, we cannot generalize from these results to individuals who have no experience of long-term sick leave. Yet, our results suggest a focus for future research.

The fact that respectful encounters were most frequent and strongest associated with feeling respected is no surprise – the whole purpose of including this item was to validate the questionnaire. Accordingly, we have not considered it in the discussion. Disrespectful encounters were used in a corresponding vein in relation to feeling wronged.

## Conclusions

A majority of patients with positive experiences from encounters with social insurance office staff also felt respected and estimated that their return to work was facilitated by these types of encounters. Feeling respected was especially associated with being listened to and being believed in. The positive effect of feeling respected was particularly manifested among patients with mental disorders. Correspondingly, a majority of those who, in addition to having experienced negative encounters, also felt wronged stated that their return to work was impeded. Feeling wronged was especially associated with being disbelieved, getting one’s condition doubted, and having one’s motivation to work questioned.

Even though encounters with social insurance office staff seem to have less effect on patients’ self-estimated ability to return to work than corresponding encounters in healthcare, the impact is not negligible. The results indicate that there is room for improving patients’ encounters on the part of social insurance office staff, in particular by listening to and believing the patients, refraining from expressing doubt regarding their conditions, and not questioning their motivation to return to work.

## Competing interests

The authors declare that they have no competing interests.

## Authors’ contributions

NL took a leading part in the conception and design of the study and in the interpretation of the results, wrote the first draft of the manuscript, and later contributed with substantial revisions. MW contributed to the conception and design of the study, contributed substantially to the statistical analysis and the interpretation of the results, and revised the manuscript for important intellectual content. DO contributed substantially to the statistical analysis and the interpretation of the results, and revised the manuscript for important intellectual content. KA designed the study questionnaire, provided data, contributed substantially to the interpretation of the results, and revised the manuscript for important intellectual content. GH contributed to the conception and design of the study, contributed substantially to the interpretation of the results and the consistency of the paper, and took a leading role in finalizing the manuscript. All authors read and approved the final manuscript.

## Pre-publication history

The pre-publication history for this paper can be accessed here:

http://www.biomedcentral.com/1471-2458/13/268/prepub
